# Radiofrequency ablation versus hepatic resection for the treatment of early-stage hepatocellular carcinoma meeting Milan criteria: a systematic review and meta-analysis

**DOI:** 10.1186/1477-7819-11-190

**Published:** 2013-08-13

**Authors:** Chenyang Duan, Mengying Liu, Zhuohang Zhang, Kuansheng Ma, Ping Bie

**Affiliations:** 1 Company Five of Cadet Brigade, Third Military Medical University, Chongqing 400038, China; 2 Institute of Hepatobiliary Surgery, Southwest Hospital, Third Military Medical University, Chongqing 400038, China; 3 Company Two of Cadet Brigade, Third Military Medical University, Chongqing 400038, China

**Keywords:** Radiofrequency ablation, Hepatic resection, Early-stage hepatocellular carcinoma, Meta-analysis

## Abstract

Current options for the treatment of the early-stage HCC conforming to the Milan criteria consist of liver transplantation, hepatic resection (HR), transcatheter arterial chemoembolization (TACE) and radiofrequency ablation (RFA) .Whether HR or RFA is the better treatment for early HCC has long been debated. The aim of our paper is to compare the therapeutic effects of radiofrequency ablation (RFA) and hepatic resection (HR) in the treatment of early-stage hepatocellular carcinoma (HCC). Controlled trials evaluating the efficacy between RFA and HR for the treatment of early-stage HCC published before June 2013 were searched electronically using MEDLINE, PubMed, Cochrane Library, and EMBASE databases. Using inclusion and exclusion criteria, two randomized controlled trials and 10 nonrandomized controlled trials were included in the meta- analysis. The results showed that the 3,5-year overall survival rates and 1,3,5 disease-free survival rates were significantly lower after RFA than after HR. However, complications after treatment were less common and the length of hospital stay was significantly shorter after RFA. Additionally, there was no significant difference in the 1-year overall survival rate between RFA and HR. The conclusions of the results show that the difference in the short-term effectiveness of RFA and HR in the treatment of small HCC is not notable, but the long-term efficacy of HR is better than that of RFA. However, HR is associated with more complications and a longer hospital stay.

## Background

Hepatocellular carcinoma (HCC) is the fifth most common malignancy cancer worldwide and the third most common cause of cancer mortality [1,2]. With the improvement of diagnostic modalities for HCC meeting the Milan criteria, defined as a single HCC ≤5 cm in the maximum diameter or up to three nodules <3 cm, the relevance ratio and detection of early-stage HCC have improved significantly [3].

Current options for the treatment of the early-stage HCC conforming to the Milan criteria consist of liver transplantation, hepatic resection (HR), transcatheter arterial chemoembolization (TACE) and radiofrequency ablation (RFA) [4-7]. Theoretically, the best treatment is liver transplantation [8-13], which offers the potential to both resect the entire potentially tumor-bearing liver and eliminate the cirrhosis. However, the limited availability of suitable living donors, high cost, as well as an increased waiting period, has raised the demand for treatment strategies of early HCC, such as HR and RFA.

Whether HR or RFA is the better treatment for early HCC has long been debated. Since the introduction of ablation for the treatment of HCC, there have been only two randomized controlled trials [14,15] and therefore the evidence of equipoise between RFA and HR is still controversial. HR has generally been accepted as the first treatment of choice for HCC in many centers. Nevertheless, the associated cirrhosis limits the extent of surgery and thus increases the risk of postoperative liver failure. RFA, which is a promising and recently developed ablation technique, was recommended as the primary treatment option for patients with early-stage HCC who are not suitable for resection or transplantation in the 2005 practice guidelines issued by the American Association for the Study of Liver Diseases [16]. It induces deep thermal injury in hepatic tissue while sparing the normal parenchyma. However, Huang and colleagues [14], Molinari and Helton [17], and Takayama and colleagues [18] reported that HR had more advantages (survival and recurrence rates) regardless of tumor size (larger or smaller than 3 cm; even smaller than 2 cm). Besides, Chen and colleagues [15], Hong and colleagues [19], Vivarelli and colleagues [20], and Montorsi and colleagues [21] concluded that RFA was as effective as HR in the treatment of solitary and small HCC.

In the current study, by performing a systematic review, we attempted to compare HR versus RFA as a primary treatment option of HCC meeting the Milan criteria.

## Methods

### Search strategy

#### ***Literature search***

Electronic searches were performed using PubMed and Medline until June 2013. The following MeSH search headings, all in English, were used: “radiofrequency ablation”, “hepatic resection”, “HCC” and “hepatocellular carcinoma”. These terms were used in different combinations. In addition, we reviewed the reference lists of the original articles and reviews on the topic to identify other potentially eligible trials. No language restrictions were made.

#### ***Data extraction and quality assessment***

Two reviewers (CD and ML) independently extracted the following parameters from each study: 1) first author and year of publication; 2) number of patients, patients’ characteristics, study design; and follow-up; 3) treatment outcomes. All relevant text, tables and figures were reviewed for data extraction. Discrepancies between the two reviewers were resolved by discussion and consensus. The quality of all selected articles was scored in accordance with the PRISMA Statement [22].

#### ***Criteria for inclusion and exclusion***

For inclusion in the meta-analysis, a study had to fulfill the following criteria: (1) all cases were diagnosed through pathology tests or more than two image logical examinations combined with clinical data comparing the initial therapeutic effects of RFA with or without TACE and HR for the treatment of early HCC, despite the etiology of liver disease, differences in viral hepatitis, or cirrhotic status; (2) no patients received any anticancer treatment before RFA or HR; (3) clearly documented indications for RFA and HR; (4) if two or more studies were reported by the same authors in the same institution, either the study of higher quality or the most recent publication was included in the analysis; (5) Child-Pugh class A or B; (6) follow-up time >3 years.

The exclusion criteria for this meta-analysis were as follows: (1) only one treatment method was used and no contrastive study was performed; (2) previously treated metastatic hepatic carcinoma or recurrent liver cancers; (3) vascular invasion, distant metastasis, or other lesions; (4) follow-up time <3 years or a small sample size (<100).

### Data analysis

Statistical analyses were performed using Review Manager Software (RevMan 5.2; Cochrane Collaboration, Oxford, UK). The continuous descriptive data of the RFA and HR groups are reported as the mean ± standard deviation, and dichotomous data are reported as the case number (n).

The Mantel-Haenszel Q-statistic was used to assess heterogeneity among the studies, and the *I*^2^ statistic was computed to examine the proportion of total variation in the study estimate due to heterogeneity. We considered *P* > 0.10 or *P* ≤ 0.10/*I*^2^ ≤ 50% to indicate no significant heterogeneity between the trials and, in such cases, a fixed effect model was selected for analysis. Conversely, we considered *P* ≤ 0.10/*I*^2^ > 50% to indicate significant heterogeneity, and a random effect model was used. In the integration results, *P* < 0.05 indicated statistical significance.

Extensive efforts were made to remove all duplicated data and include all studies published to date. Publication bias in outcomes was assessed and treated using standard methodology. Funnel plots were used to visually inspect the relationship between sample size and treatment effects for the two groups.

## Results

### Search results

**Figure 1:**
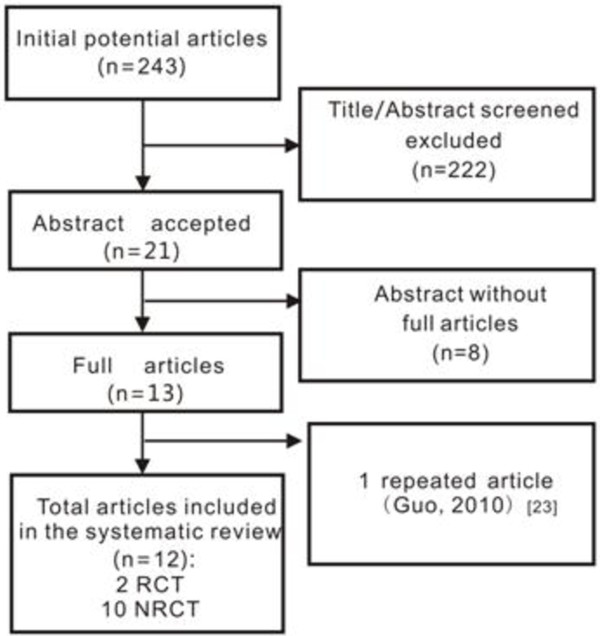
**Process of study selection.** NRCT, nonrandomized controlled trial; RCT, randomized controlled trial.

A total of 243 relevant articles were identified in a combined search of MEDLINE, PubMed, Cochrane Library, and EMBASE databases covering studies published before June 2013 and a manual approach (search of studies cited in previous reviews and of reference lists from the identified articles). In total, 222 articles were excluded after scanning the title/abstract because they were not relevant to the purpose of this meta-analysis, and full-length articles could not be obtained for eight references. One duplicate article from the same author was excluded [23]. Ultimately, 12 articles were included in the systematic review, including two randomized controlled trials and 10 nonrandomized controlled trials (Figure 1).

A total of 8,612 subjects were included in the meta-analysis: 4,295 patients who were treated with RFA as the initial treatment and 4,279 patients who underwent HR. The follow-up auxiliary examinations included radiographic tests, such as ultrasound, computed tomography or magnetic resonance imaging, in combination with physical examination. The largest study included 5,879 patients [11], and the smallest study included 100 patients [24]. The characteristics of the 12 studies included in the meta-analysis are listed in Table 1.

**Table 1:** Characteristics of the studies included in the meta-analysis

**Study**	**Research period**	**RFA (n)**	**HR (n)**	**Trial type**	**Tumor diameter (cm)**	**Grade**
Chen *et al.* 2006 [15]	1999–2004	71	90	RCT	≤5	A
Cho *et al.* 2005 [25]	2000–2002	99	61	NRCT	≤5	B
Guglielmi *et al.* 2008 [26]	1996–2006	109	91	NRCT	≤3	B
Guo *et al.* 2013 [27]	2002–2007	94	102	NRCT	≤5	B
Hasegawa *et al.* 2008 [28]	2000–2003	3022	2857	NRCT	≤3	B
Hiraoka *et al.* 2008 [29]	2000–2007	105	59	NRCT	3–5	B
Hong *et al.* 2005 [19]	2000–2003	55	55	NRCT	≤4	B
Huang *et al.* 2011 [14]	2000–2005	413	648	NRCT	≤3	B
Lu *et al.* 2006 [30]	2002–2005	51	54	RCT	≤3	A
Lupo *et al.* 2007 [24]	2003–2004	42	60	NRCT	3–5	B
Ueno *et al.* 2009 [31]	2000–2005	155	123	NRCT	≤5	B
Vivarelli *et al.* 2004 [20]	1998–2005	79	79	NRCT	≤5	B

*HR* hepatic resection, *NRCT* nonrandomized controlled trial, *RCT* randomized controlled trial, *RFA* radiofrequency ablation.

### Meta-analysis

We mainly compared the following eight indicators between RFA and HR in the treatment of small HCC: 1-, 3-, and 5-year overall survival rates; 1-, 3-, and 5-year disease-free rates; post-treatment complications; and hospital stay.

#### ***One-year overall survival rate***

**Figure 2:**
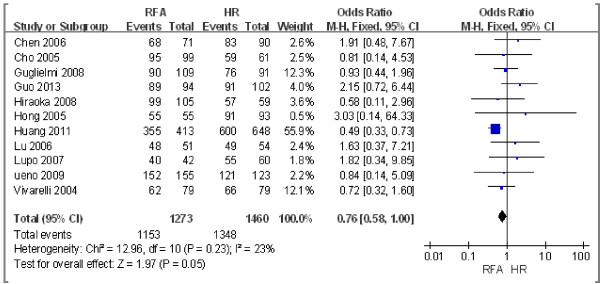
**Comparison of the 1-year overall survival rate between radiofrequency ablation (RFA) and hepatic resection (HR).** CI, confidence interval; M-H, Mantel-Haenszel.

Eleven studies including 2,733 patients [14,15,19,20,24-27,29-31] compared the 1-year overall survival rate after RFA and HR. Using the odds ratio (OR) as an indicator, we used the χ^2^ test to examine heterogeneity. The result was *P* = 0.23/*I*^2^ = 23%, which indicated that there was no heterogeneity between the two groups. Therefore, we used a fixed effect model to perform the meta-analysis, the results of which were as follows: OR = 0.76; 95% confidence interval (CI) = 0.58 to 1.00; *P* = 0.05. These findings indicated that there were no differences in the 1-year overall survival rate between RFA and HR (Figure 2).

#### ***Three-year overall survival rate***

**Figure 3:**
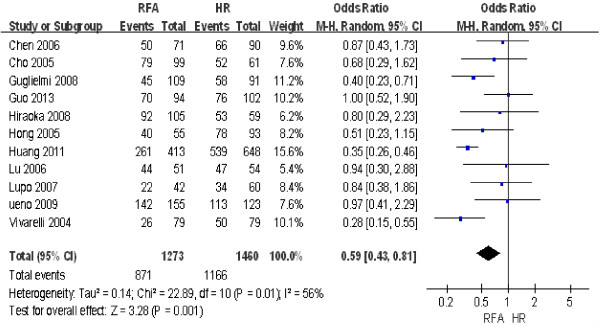
**Comparison of the 3-year overall survival rate between radiofrequency ablation (RFA) and hepatic resection (HR).** CI, confidence interval; M-H, Mantel-Haenszel.

The same 11 studies were also used to compare the 3-year overall survival rate between RFA and HR. The findings of the heterogeneity test were *P* = 0.01/*I*^2^ = 56%, which indicated that there was significant heterogeneity between the two groups. Consequently, we used a random effect model for the analysis, the results of which were as follows: OR = 0.59; 95% CI = 0.43 to 0.81; *P* = 0.001. Thus, the 3-year overall survival rate after HR was significantly higher than that after RFA (Figure 3).

#### ***Five-year overall survival rate***

**Figure 4:**
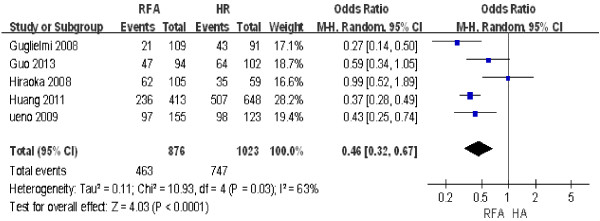
**Comparison of the 5-year overall survival rate between radiofrequency ablation (RFA) and hepatic resection (HR).** CI, confidence interval; M-H, Mantel-Haenszel.

Five studies including 1,899 patients [14,26,27,29,31] were used to compare the 5-year overall survival rate after RFA and HR. The results of the heterogeneity test were *P* = 0.03*/I*^2^ = 63%, indicating significant heterogeneity between the two groups. Therefore, we used a random effect model, the results of which were as follows: OR = 0.46; 95% CI = 0.32 to 0.67; *P* < 0.0001. These findings indicated that the 5-year overall survival rate after HR was significantly higher than that after RFA (Figure 4).

#### ***One-year disease-free survival rate***

**Figure 5:**
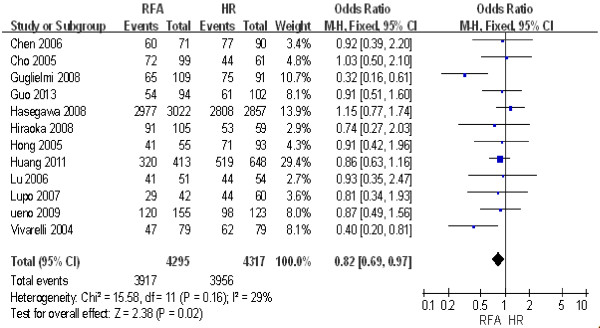
**Comparison of the 1-year disease-free survival rate between radiofrequency ablation (RFA) and hepatic resection (HR).** CI, confidence interval; M-H, Mantel-Haenszel.

Twelve studies including 8,612 patients [14,15,19,20,24-31] were used to compare the 1-year disease-free survival rate after RFA and HR. The results of the heterogeneity test were *P* = 0.16/*I*^2^ = 29%, indicating no heterogeneity between the two groups. Therefore, we used a fixed effect model for the meta-analysis, the results of which were as follows: OR = 0.82; 95% CI = 0.69 to 0.97; *P* = 0.02. These findings revealed that the 1-year disease-free survival rate after HR was significantly higher than that after RFA (Figure 5).

#### ***Three-year disease-free survival rate***

**Figure 6:**
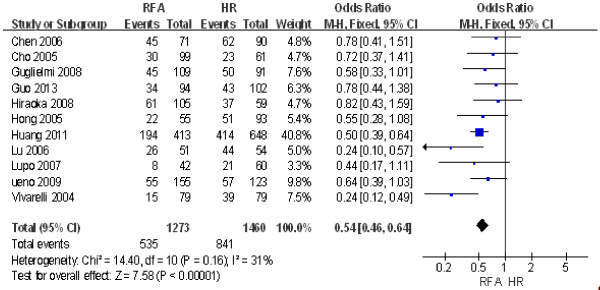
**Comparison of the 3-year disease-free survival rate between radiofrequency ablation (RFA) and hepatic resection (HR).** CI, confidence interval; M-H, Mantel-Haenszel.

The same 12 studies were used to compare the 3-year disease-free survival rate between RFA and HR. The results of the heterogeneity test were *P* = 0.16/*I*^2^ = 31%, indicating no heterogeneity between the two groups. Therefore, we used a fixed effect model for the meta-analysis, the results of which were as follows: OR = 0.59; 95% CI = 0.43 to 0.81; *P* = 0.001. These findings indicated that the 3-year disease-free survival rate after HR was significantly higher than that after RFA (Figure 6).

#### ***Five-year disease-free survival rate***

**Figure 7:**
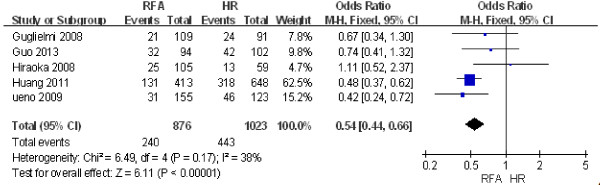
**Comparison of the 5-year disease-free survival rate between radiofrequency ablation (RFA) and hepatic resection (HR).** CI, confidence interval; M-H, Mantel-Haenszel.

Five studies including 1,899 patients [14,26,27,29,31] were used to compare the difference in the 5-year disease-free survival rate between RFA and HR. The results of the heterogeneity test were *P* = 0.17*/I*^2^ = 38%, indicating no heterogeneity between the two groups. Therefore, we used a fixed effect model for the meta-analysis, the results of which were as follows: OR = 0.54; 95% CI = 0.44 to 0.66; *P* < 0.00001. These data revealed that the 5-year disease-free survival rate after HR was significantly higher than that after RFA (Figure 7).

#### ***Complications after treatment***

**Figure 8:**
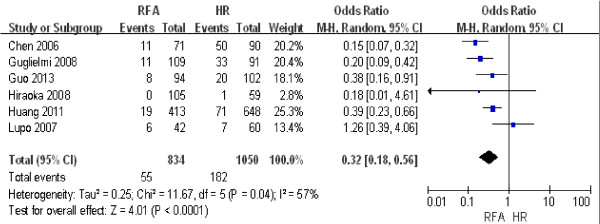
**Comparison of the number of complications between radiofrequency ablation (RFA) and hepatic resection (HR).** CI, confidence interval; M-H, Mantel-Haenszel.

The complications after treatment included gastrointestinal bleeding, ascites, serious infection, biliary duct injury, jaundice, hepatic failure, and death. Six studies including 1,782 patients [14,15,24,26,27,29] were used to compare the difference in the number of complications between RFA and HR. The results of the heterogeneity test were *P* = 0.04/*I*^2^ = 57%, indicating significant heterogeneity between the two groups. Therefore, we used a random effect model for the meta-analysis, the results of which were as follows: OR = 0.32; 95% CI = 0.18 to 0.56; *P* < 0.0001. These results revealed that more complications occurred after HR than after RFA (Figure 8).

#### ***Hospital stay***

**Figure 9:**
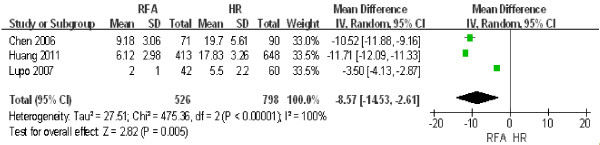
**Comparison of the length of hospital stay between radiofrequency ablation (RFA) and hepatic resection (HR).** CI, confidence interval; M-H, Mantel-Haenszel.

Three studies including 1,324 patients [14,15,24] were used to compare the difference in hospital stay between RFA and HR. The results of the heterogeneity test were *P* < 0.00001/*I*^2^ = 100%, indicating significant heterogeneity between the two groups. Therefore, we used a random effect model for the analysis, the results of which were as follows: OR: -8.57; 95% CI = -14.53 to -2.61; *P* = 0.005. These data indicated that the length of hospital time was significantly longer after HR than after RFA (Figure 9).

#### ***Sensitivity analysis and publication bias***

**Figure 10:**
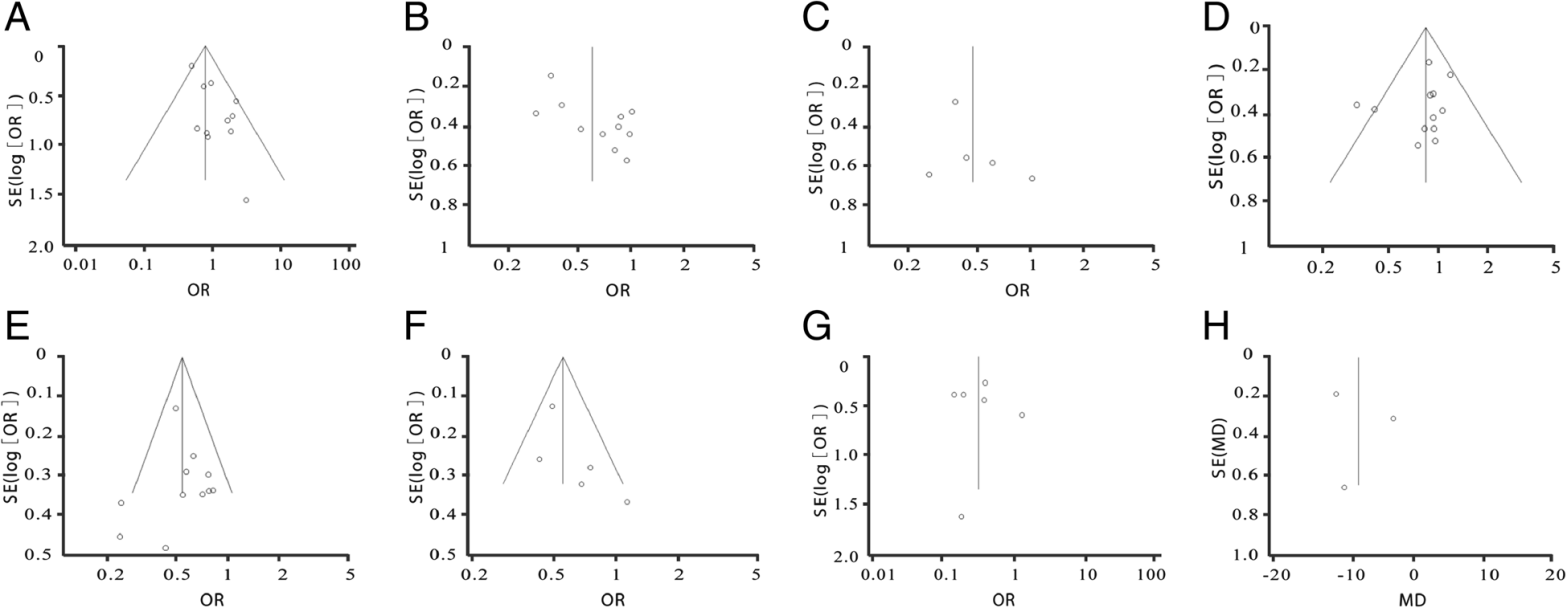
**Funnel plot for each comparison. (A)** 1-year overall survival rate; **(B)** 3-year overall survival rate; **(C)** 5-year overall survival rate; **(D)** 1-year disease-free survival rate; **(E)** 3-year disease-free survival rate; **(F)** 5-year disease-free survival rate; **(G)** complications after treatment; **(H)** hospital stay. OR, odds ratio.

We used fixed and random effect models to test each indicator, and the results were correlated. We created a funnel plot for each comparison (Figure 10). These eight plots were basically inverted and funnel-shaped with bilateral symmetry, indicating a lack of publication bias and reliable conclusions.

## Discussions

Currently, several treatment methods have been developed for HCC, including liver transplantation, hepatic resection, RFA, microwave therapy, TACE, and molecular targeted drug therapy [32]. HR has always been regarded as the traditional first-line treatment for small HCC. With the development of RFA, this modality may become the first-line treatment for HCC. Therefore, many controlled trials have compared HR and RFA in the treatment of small HCC. To verify the effectiveness and safety of these treatments, it is necessary to perform meta-analysis of these trials; this meta-analysis may also provide a basis for evidence-based medicine.

RFA is a medical procedure in which part of the tumor is ablated using the heat generated from a high-frequency alternating current under image guidance (such as ultrasound, computed tomography or magnetic resonance imaging). Currently, RFA is mainly used for primary hepatic carcinoma that cannot be resected [33], metastatic hepatic carcinoma [34], and recurrent hepatic carcinoma after surgery [35], as well as for patients who are unwilling to undergo HR. RFA has been regarded as a suitable treatment for HCC because of its low trauma, low number of complications, and strong repeatability [36].

Meta-analysis can be used to evaluate the efficacy of RFA and HR in treating small HCC in patients with tumor diameters ≤5 cm. In this meta-analysis, no difference was noted between HR and RFA regarding the 1-year overall survival rate. However, HR was associated with higher 3- and 5-year overall survival rates. Additionally, the 1-, 3-, and 5-year disease-free rates were significantly higher after HR than after RFA. The results of this meta-analysis are also confirmed by two Markov models [17,37].

This finding may be explained by the fact that RFA is primarily directed at primary tumor lesions, but some satellite lesions may be missed. In contrast, HR can be used to resect the primary lesions as well as satellite lesions transferred through portal vein branches. Moreover, factors such as the shape and distribution of the tumor and range of ablation have a much stronger effect on RFA than on HR.

Another comparison revealed that RFA is associated with fewer complications and a shorter hospital stay, indicating that RFA is relatively safe and noninvasive.

Our study findings revealed that RFA is a safe and effective modality for treating early-stage HCC. However, considering the better survival rate after HR and its ability to prevent recurrence, HR has an irreplaceable role in the treatment of HCC, which makes it the first-line treatment for this malignancy.

The limitation of our study was that only two randomized controlled trials were included. Therefore, we expect that more researchers will perform large, well-designed randomized controlled trials to clarify which treatment is most effective against HCC.

## Abbreviations

CI: Confidence interval; HCC: Hepatocellular carcinoma; HR: Hepatic resection; OR: Odds ratio; RFA: Radiofrequency ablation; TACE: Transcatheter arterial chemoembolization.

## Competing interests

The authors declare that they have no competing interests.

## Authors’ contributions

CD independently searched references and extracted the parameters from each study, took charge of data statistics and drafted the manuscript. ML independently searched references and extracted the parameters from each study. ZZ participated in the discussion of the discrepancies between the two reviewers. KM provided the National Science Foundation of China and revised the manuscript. PB participated in the manuscript revision. All authors read and approved the final manuscript.
